# The Oncogene Addiction Switch from NOTCH to PI3K Requires Simultaneous Targeting of NOTCH and PI3K Pathway Inhibition in Glioblastoma

**DOI:** 10.3390/cancers11010121

**Published:** 2019-01-20

**Authors:** Norihiko Saito, Nozomi Hirai, Kazuya Aoki, Ryo Suzuki, Satoshi Fujita, Haruo Nakayama, Morito Hayashi, Keisuke Ito, Takatoshi Sakurai, Satoshi Iwabuchi

**Affiliations:** Department of Neurosurgery, Toho University Ohashi Medical Center, Tokyo 153-8515, Japan; nozomi.hirai@med.toho-u.ac.jp (N.H.); kaoki@med.toho-u.ac.jp (K.A.); ryo.suzuki@med.toho-u.ac.jp (R.S.); satoshi.fujita@med.toho-u.ac.jp (S.F.); haruonakayama@med.toho-u.ac.jp (H.N.); morito@med.toho-u.ac.jp (M.H.); keisuke@med.toho-u.ac.jp (K.I.); cherry@med.toho-u.ac.jp (T.S.); iwabuchi@med.toho-u.ac.jp (S.I.)

**Keywords:** NOTCH, glioma initiating cell, PTEN, PI3K pathway, glioblastoma

## Abstract

The NOTCH pathway regulates neural stem cells and glioma initiating cells (GICs). However, blocking NOTCH activity with γ-secretase inhibitors (GSIs) fails to alter the growth of GICs, as GSIs seem to be active in only a fraction of GICs lines with constitutive NOTCH activity. Here we report loss of *PTEN* function as a critical event leading to resistance to NOTCH inhibition, which causes the transfer of oncogene addiction from the NOTCH pathway to the PI3K pathway. Drug cytotoxicity testing of eight GICs showed a differential growth response to GSI, and the GICs were thus stratified into two groups: sensitive and resistant. In the sensitive group, GICs with loss of PTEN function appeared less sensitive to GSI treatment. Here we show that NOTCH regulates PTEN expression and the activity of the PI3K pathway in GICs, as treatment with GSI attenuated the NOTCH pathway and increased PTEN expression. NOTCH regulates PTEN expression via Hes-1, as knockdown of Notch or Hes1 increased expression of PTEN. This novel observation suggests that both pathways must be simultaneously inhibited in order to improve therapeutic efficacy in human glioblastomas (GBMs).

## 1. Introduction

Glioblastoma (GBM), the most common adult glioma, has a poor prognosis. Genetic heterogeneity between patients and even within tumors is high, and GBM is characterized by evolving genetic aberration resulting from dynamic genetic instability. The genes most commonly affected are *CDKN2A*, *TP53*, *EGFR*, *PTEN* and *RB* [1]. By combining sequencing data with other types of genomic information, the Cancer Genome Atlas team produced a tentative overview of the main biological pathways involved in GBM. Each of the three pathways (namely, the CDK/RB, p53 and RTK/RAS/PI3K pathways) was disrupted in more than three-quarters of GBM tumors. Signal transduction pathways are complex and exhibit overlap and crosstalk [2]. The complexity of these pathways may allow for compensatory effects in alternative pathways, which could lead to resistance to single agents that regulate only one target. Successful novel therapeutic strategies for GBMs may thus require simultaneous targeting of multiple dysregulated molecules.

The NOTCH signaling pathway is an evolutionarily conserved system that is important in most multicellular processes such as neural differentiation, proliferation, survival, angiogenesis and stemness [3,4,5]. About 45% of proneural GBMs exhibit a high expression of representative NOTCH pathway genes, which has been implicated in the pathogenesis of solid tumors [6]. When the NOTCH receptor is triggered by a ligand, it promotes two proteolytic cleavage events at the NOTCH receptor: by means of an ADAM metalloprotease and γ-secretase complex. The cleavage can release the NOTCH intracellular domain (NICD), which translocates to the nucleus and interacts with the CSL-binding protein to activate expressions of NOTCH targeting genes [3,4]. 

Recent studies suggest that PTEN is regulated through the NOTCH pathway in a variety of settings, such as fibroblasts [7,8], T-cell acute lymphoblastic leukemia cells [9] and prostate tumor cells [10]. NOTCH interaction with PTEN has been well characterized in T-cell leukemia, in which NOTCH and PTEN induce resistance to γ-secretase inhibition. Here we report that PTEN regulates GBM sensitivity to γ-secretase inhibitors (GSIs), thereby highlighting the need for simultaneous inhibition of the PI3K/AKT and NOTCH pathways in PTEN-mutant GBMs. Thus, PTEN may be an important factor of GSI-induced attenuation of cell growth through a regulatory circuit linking NOTCH signaling with PTEN expression. This finding supports a need for combination therapeutic strategies in the treatment of GBM.

## 2. Results

### 2.1. GICs Show Differential Growth in Response to GSIs

**Figure 1:**
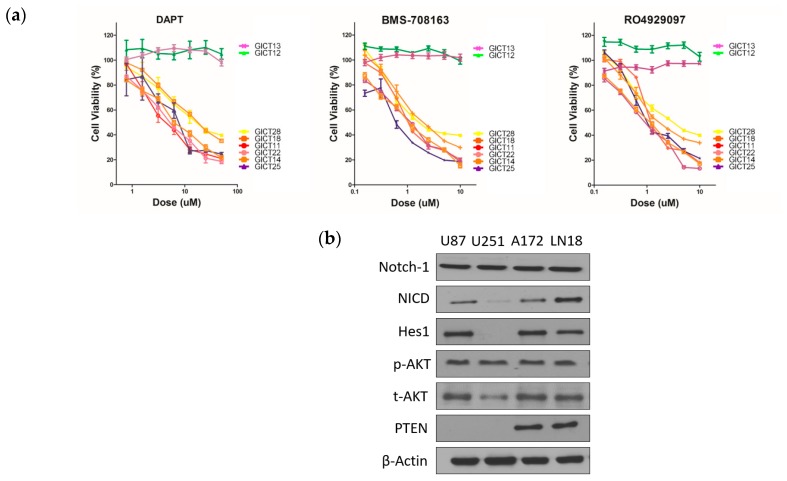

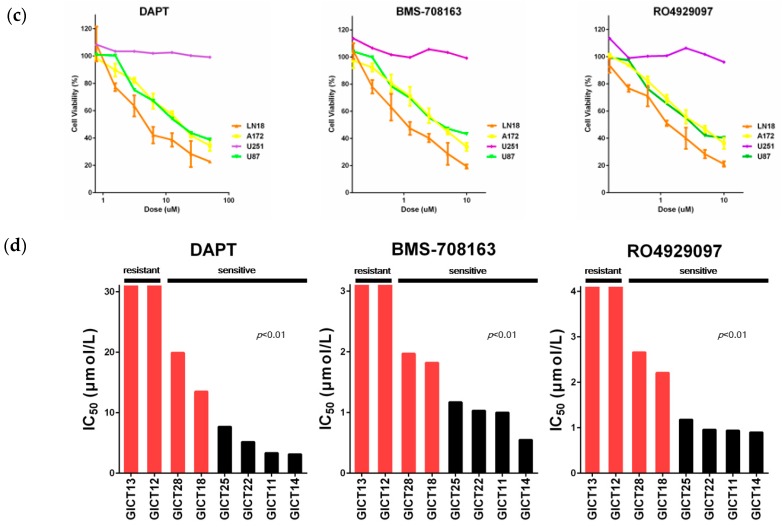
γ-Secretase inhibitors (GSIs) showed dose-dependent growth inhibition of glioma tumor-initiating cells (GICs). (**a**) A panel of GIC lines was treated with various concentrations of the GSIs. Cells were treated with increasing concentrations of GSIs in triplicate wells for 72 h, and cell viability was assessed with the CellTiter-Blue assay. Cell viability in the vehicle control was considered to be 100%; (**b**) GSIs showed dose-dependent growth inhibition of glioma cells. A panel of glioma cell lines was treated with various concentrations of the GSIs. Cells were treated with increasing concentrations of GSIs in triplicate wells for 72 h, and cell viability was assessed with the CellTiter-Blue assay. Cell viability in the vehicle control was considered to be 100%; (**c**) Western blotting of the Notch signaling, AKT and PTEN in glioma cell lines. β-Actin was used as loading control; (**d**) Waterfall plot of IC50 values for eight GICs. These figures show that GSIs have a particular growth inhibition signature: some cell lines are very sensitive and others are relatively resistant.

We quantified sensitivity to three GSIs, as seen in Figure S1, in a panel of eight glioma initiating cell lines (GICs) and four glioma cell lines by measuring the IC50 or half-maximal inhibitory concentration after 72 h of continuous exposure. GSIs showed a dose-dependent growth inhibition of GICs and glioma cell lines (Figure 1a,b). Expression of the Notch signaling, PTEN and AKT are shown in Figure 1c [11]. NICD and Hes1—a NOTCH-1 pathway component—were expressed in U87, A172 and LN18. PTEN expression was absent in U87 and U251, suggesting that loss of PTEN function (Figure 1c). Figure 1d shows representative waterfall plots of the differential responses to GSIs, which were used to classify GICs as sensitive and resistant. Sensitive cell lines were those with IC50 values of 3–18 μmol/L for N-[N-(3,5-difluorophenacetyl)-L-alanyl]-S-phenylglycine t-butyl ester (DAPT) and 0.5–2 μmol/L for BMS-708163 and RO4929097. Resistant cell lines were those with IC50 values greater than 20 μmol/L for DAPT and greater than 3 μmol/L for BMS-708163 and RO4929097 (Figure 1d).

### 2.2. Expression of the NOTCH Signaling Pathway and PTEN Status in GICs

**Figure 2:**
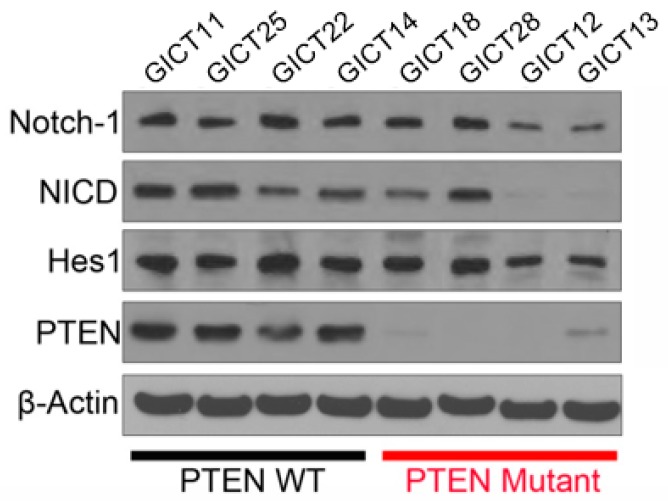
Western blotting of the NOTCH signaling pathway in eight GICs. Activated Notch-1 (NICD) and Hes1 were expressed in sensitive GICs. β-Actin was used as the loading control.

To identify associations between the activation of the NOTCH signaling pathway, PTEN status and the sensitivity of GSIs, Western blot analysis was used to evaluate gene expression in the NOTCH signaling pathway in eight GIC lines, as seen in Figure 2. NICD and Hes1—a NOTCH-1 pathway component—were all expressed in sensitive and resistant GICs. PTEN expression was absent, or very low, in resistant GICs, which suggests that the response may be related to PTEN status in GICs.

### 2.3. NOTCH Regulates Activity of the PI3K/AKT Pathway via Hes1 in PTEN–Wild-Type GICs

**Figure 3:**
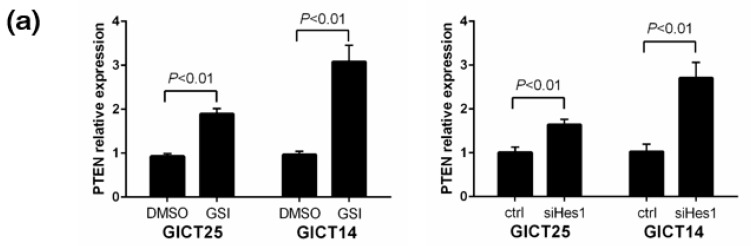

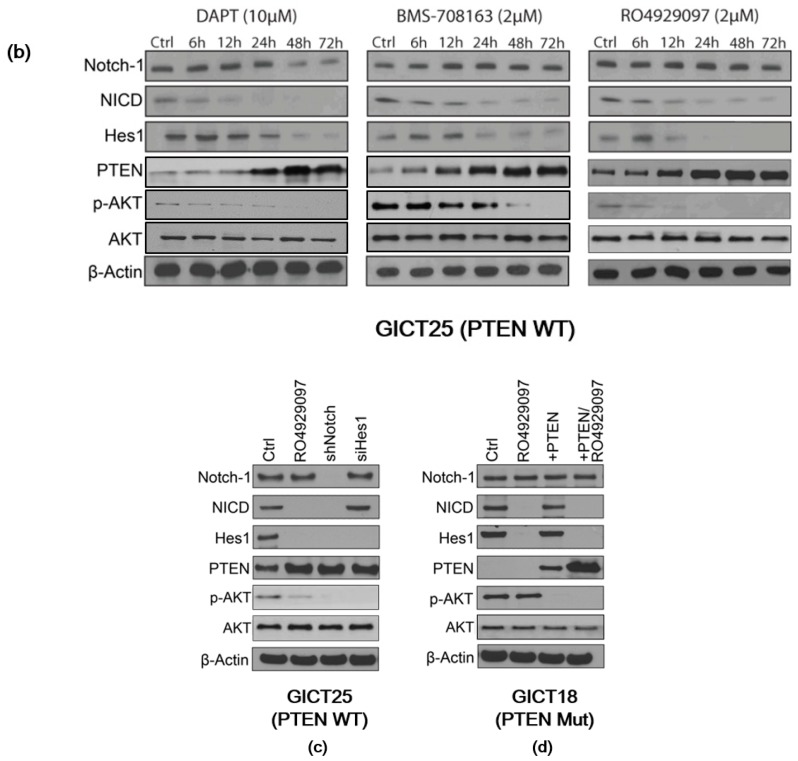
(**a**) Real-time PCR analysis of PTEN transcript levels upon NOTCH-1 inhibition by GSI in GICT25 and GICT14 relative to (DMSO) controls. GAPDH levels were used as the reference control; (**b**) A GICT25 panel was treated with the indicated doses of DAPT, BMS-708163 and RO4929097 for the indicated time intervals. All GSIs inhibited the expression of NICD, Hes1 and p-AKT in a time-dependent manner. The decrease in NICD was followed by a decrease in Hes1 expression, whereas Notch-1 expression did not change; (**c**) Western blotting confirmed the knockdown effect of Notch shRNA lentivirus on GICT25. siRNA knockdown of Hes1 decreased HES1 expression and increased PTEN expression; (**d**) After transfection with the PTEN gene, PTEN expression increased with GSI treatment.

The close association between the presence of PTEN mutations and GSI resistance in GICs prompted us to ask whether PTEN might be functionally linked to NOTCH-1 signaling. Real-time PCR analysis of PTEN transcript levels upon NOTCH-1 inhibition by GSI showed PTEN relative expression levels were increased by GSI treatment in GICT25, i.e., GSI-sensitive/PTEN–wild-type cells (Figure 3a). In GICT25, NICD and HES1 expressions decreased and PTEN expression increased after treatment with GSIs, as seen in Figure 3b. In NOTCH knockdown experiments, NOTCH-1, NICD and HES1 expressions were decreased and PTEN expression was increased. siRNA knockdown of Hes1 decreased HES1 expression and increased PTEN expression, thus suggesting that Hes1 caused the down-regulation of PTEN expression by transcriptional repression of the PTEN promoter (Figure 3c). In GICT18—a PTEN-mutant GIC—Western blotting was used to investigate the expression of NICD, HES1, PTEN, p-AKT and total AKT proteins. Transfection with PTEN resulted in the up-regulation of PTEN expression and down-regulation of p-AKT expression in GICT18. After transfection with PTEN, PTEN expression increased after GSI treatment, as seen in Figure 3d. These results indicate that GSI might affect the PTEN and AKT pathway and that PTEN is a downstream target of the NOTCH pathway via HES1 in PTEN–wild-type GICs. 

### 2.4. The Combination of GSI and PI3K Inhibitor had a Synergistic Effect in PTEN Mutant GICs

**Figure 4:**
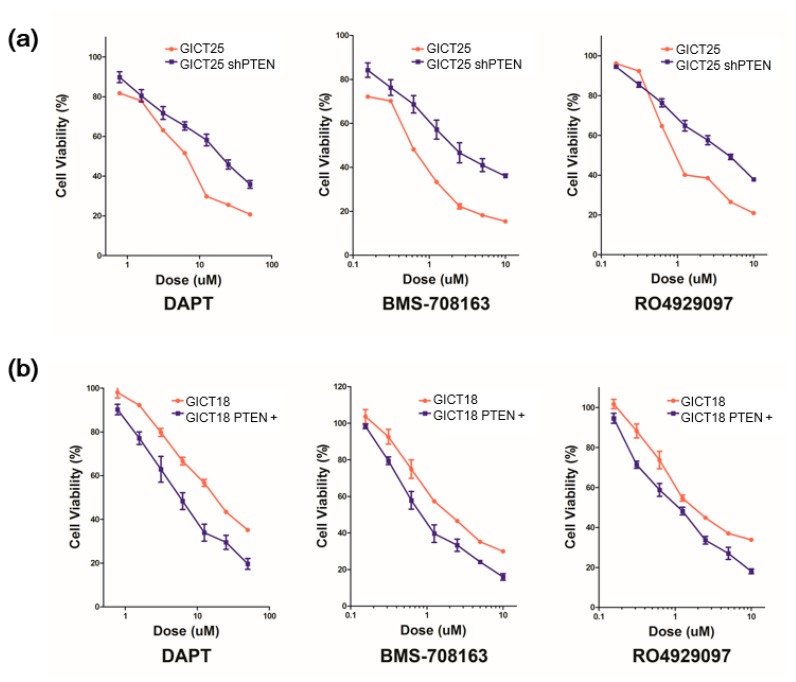
PTEN expression is required for the GSI response in cell growth inhibition. (**a**) PTEN knockdown decreased the response to GSI in PTEN–wild-type GIC; (**b**) PTEN expression increased the response to GSI in PTEN-mutant GIC.

**Figure 5:**
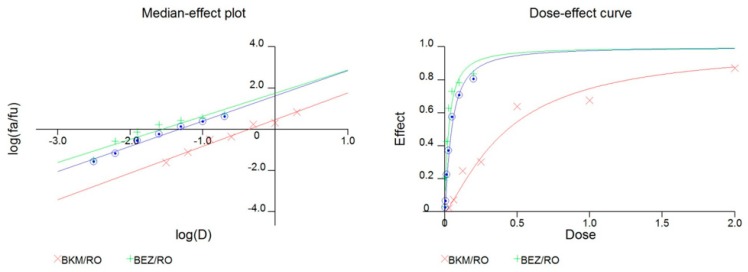
The combination of a GSI and PI3K inhibitor had a synergistic effect on PTEN-mutant GIC.

We performed drug sensitivity testing and compared the control with PTEN knockdown in GICT25. The IC50 of GICT25 increased from 7.69 to 19.8 μM in DAPT, from 0.55 to 2.00 μM in BMS-708163 and from 0.96 to 4.67 μM in RO4929097, indicating a decrease in sensitivity to GSI treatment, as seen in Table 1a and Figure 4a. We also performed drug sensitivity testing and compared the control with PTEN-transfected GICT18. The IC50 of GICT18 decreased from 13.50 to 5.78 μM in DAPT, from 1.82 to 0.84 μM in BMS-708163 and from 2.21 to 1.11 μM in RO4929097, indicating an increase of sensitivity to GSI treatment, as seen in Table 1b and Figure 4b. We tested the efficacy of combining RO4929097 with two inhibitors of the PI3K/AKT/mTOR pathway, namely BEZ235 and BKM120. For BKM120 and RO4929097, the maximum concentration was 2 μM for BKM120 and 1 μM for RO4929097, which were diluted to a 2:1 ratio. For BEZ235 and RO4929097, the maximum concentration was 200 nM for BEZ235 and 1 μM for RO4929097, which were diluted to a 1:5 ratio. We calculated the Combination Index by using the Calcusyn software package and observed the synergistic inhibition of cell proliferation in GICT18, as seen in Figure 5 and Table 2.

### 2.5. The Combination of GSI and PI3K Inhibitor Regulated Survival in an Orthotropic Mouse Model

**Figure 6:**
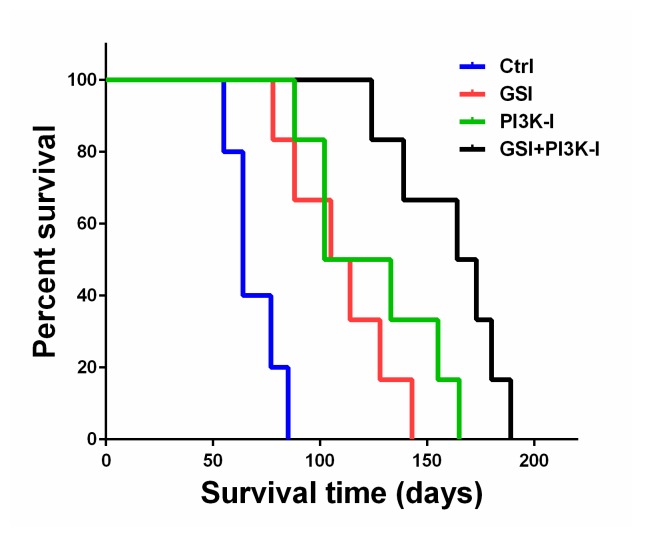
The combination of a GSI and PI3K inhibitor had a synergistic effect in PTEN-mutant GIC. Kaplan–Meier survival probability plots of tumor-bearing mice in vehicle or RO4929097, BKM120 and combined RO4929097 and BKM120 treatment groups were graphed, and the log-rank test was used to compare groups. All treatments (different colored lines) showed a statistically significant improvement versus control (*p* < 0.05).

To assess the anti-glioma efficacy of the combination of GSI and PI3K inhibitor in vivo, we used a GICT25 orthotopic model of human glioma intracranial xenografts in nude mice. On day 4 after tumor cell implantation, the animals were treated with vehicle or with 10 mg/kg RO4929097 in methyl cellulose alone, 20 mg/kg BKM120 in methyl cellulose alone, a combination of RO4929097 and BKM120 in methyl cellulose or methyl cellulose alone (control) once a week for a total of 5 weeks, as described in the Materials and Methods section. The median survival duration for animals treated with methylcellulose (control) was 64 days, as seen in Figure 6. Treatment with 10 mg/kg RO4929097 and 20 mg/kg BKM120 alone extended survival to a median of 109.5 and 117.5 days, respectively (*p* < 0.05, log-rank test for both experiments). Combination treatment with RO4929097 and BKM120 significantly increased the survival of mice, as compared with either agent alone (median 168.5 days, *p* < 0.05). These results show that the combination of RO4929097 and BKM120 can inhibit tumor growth and prolong the survival of mice with xenograft tumors, which suggests that the combination of RO4929097 with BKM120 has potential therapeutic efficacy in vivo.

## 3. Discussion

The NOTCH signaling pathway is involved in cell-fate decisions during normal development and in multicellular processes and has been implicated in the maintenance of neural progenitors during brain development [12,13]. In gliomas, NOTCH signaling seems to confer radioresistance to glioma initiating cells [14] and the inhibition of NOTCH through GSIs [15] or Delta-4 monoclonal antibodies [16] decreased the numbers of glioma initiating cells and/or their tumorigenicity in some preclinical models. This suggests that a therapeutic strategy that includes NOTCH inhibitors might be used clinically to target glioma initiating cells and overcome chemoresistance and radioresistance. We previously reported that proneural GICs with high NOTCH pathway activation responded to GSIs and that PTEN status was an important factor in the sensitivity to GSI treatment [6]. Amplifying or activating mutations of PIK3CA were found in about 15% of patients with GBM [17,18,19]. Similarly, loss-of-function mutations, chromosomal deletions or epigenetic gene silencing of PTEN, which are associated with poor survival [19], were found in approximately 40% of GBM cases [1].

The close association between PTEN mutation and GSI resistance in GICs suggests that PTEN might be linked to NOTCH signaling functionally. Analysis of the transcriptional responses of GSI-sensitive GICs with PTEN wildtype to NOTCH inhibition showed significant upregulation of the PTEN expression level. However, the mechanism responsible for PI3K-AKT upregulation via NOTCH activation in GICs remains unknown. The inhibitory effect of NOTCH signaling on PTEN expression is inconsistent with the established role of NOTCH as a transcriptional activator [20,21,22,23]. Thus, we hypothesized that the inhibition of PTEN by NOTCH could be mediated by HES1 regulated by NOTCH1. 

“Oncogene addiction” refers to the need that tumor cells have for sustained abundant oncogene signaling, which maintains their viability [24,25,26], and explains the requirement for continuous NOTCH signaling in GICs with mutations in NOTCH [27,28]. Our data indicate that loss of function with PTEN in GICs may result in resistance to NOTCH inhibition by GSI treatment. Thus, we hypothesized that GSI resistance results from a change in oncogene addiction, from NOTCH to constitutive AKT signaling. Our preliminary data reveal a synergistic attenuation of cell growth by the combination of GSIs and PI3K inhibitors in PTEN mutant GICs. Thus, a future study should evaluate the efficacy of combined NOTCH and PI3K/AKT inhibition therapies in glioma.

## 4. Materials and Methods

### 4.1. Cell Lines and Reagents

The four glioma cell lines used in this study were obtained from the JCRB cell bank. The eight glioma initiating cell lines were maintained in neurosphere medium by using a previously described method [29] to isolate neurosphere-forming cells from surgical specimens of human GBM. The study was approved by the Institutional Review Board of Toho University (H22-62). These GIC lines were cultured as GBM neurospheres in DMEM/F12 medium supplemented with B27 (Invitrogen, Grand Island, NY, USA), L-glutamine (GIBCO), penicillin/streptomycin and growth factors (20 ng/mL EGF and 20 ng/mL FGF-2; Invitrogen). DAPT, BMS-708163, RO4929097 and NVP-BKM120 were purchased from Selleck Chemicals. For in vitro use, all inhibitors were dissolved in dimethyl sulfoxide (DMSO; Sigma-Aldrich, St. Louis, MO, USA) to a concentration of 10 mmol/L, stored at −20 °C and further diluted to an appropriate final concentration in DMEM/F12 medium at the time of use. The DMSO in the final solution did not exceed 0.1% (v/v).

### 4.2. Cell Proliferation Assay

Cells were seeded in 96-well plates (2000 cells/well) and incubated at 37 °C for 24 h before addition of serial dilutions of GSIs DAPT, BMS-708163 and RO4929097. Growth inhibition was estimated using the CellTiter-Blue (Promega, Madison, WI, USA) viability assay. The IC50 value was calculated as the mean drug concentration required in order to inhibit cell proliferation by 50% compared with vehicle controls. The data are expressed as percentages of the vehicle-treated controls, and IC50 values were calculated with CalcuSyn 2.0 software (BIOSOFT, Great Shelford, Cambridge, UK).

### 4.3. Western Blot Analysis

Cells were harvested in lysis solution, as previously described [30], and subjected to Western blotting. Membranes were probed with the following primary antibodies: NOTCH-1, PTEN, phospho-specific AKT, total AKT (Cell Signaling, Boston, MA, USA) and Hes-1 (Millipore, Burlington, MA, USA). Anti–β-actin antibody was purchased from Sigma and used as a loading control.

### 4.4. RNA Extraction and cDNA Synthesis

Total RNAs were extracted from each sample and placed in 350 μL of RLT buffer (Qiagen, Hilden, Germany) supplemented with 1% β-mercaptoethanol. Next, the total RNAs were purified with an RNeasy Micro Kit (Qiagen, Valencia, CA, USA) according to the manufacturer’s protocol. After RNA extraction, we synthesized first-strand cDNA by using random primers and TaqMan reverse-transcription reagents (Applied Biosystems, Foster City, CA, USA).

### 4.5. Quantitative Real-Time PCR Analysis

Quantitative real-time PCR was performed with an ABI PRISM 7000 sequence detection system (Applied Biosystems) according to the manufacturer’s instructions. The relative expressions of PTEN mRNAs were normalized to the amount of GAPDH in the same cDNA by using the delta Ct method, as described by the manufacturer. Each sample was assayed in triplicate and analyzed with SDS software (Applied Biosystems). 

### 4.6. Knockdown of Notch-1 by Lentiviral shRNA

Lentiviral vector encoding shRNA for PTEN was purchased from GeneCopoeia Inc. Lentiviral vector with pLKO.1-mediated expression of shRNA for targeting human PTEN was performed according to the manufacturer’s instructions. Lentiviral particles, which expressed targeting and control scramble, were produced in HEK293 cells with the mixed set of packing plasmids, and the viruses were concentrated and titered as previously described [31]. GICT25 cells were infected with the PTEN shRNA. The produced lentiviruses were concentrated with a Centricon Plus-20 centrifugal filter device (Millipore). To ensure that the same number of lentiviral particles was used in each experiment, the produced lentiviral stock was stored at −80 °C. For the in vitro infection of GICs with the lentivirus, we disaggregated cultured tumorspheres before infection, to increase infection efficiency and uniformity. To infect target cells with lentiviruses, we exposed GICT25 for 24 h. Cells were washed and then cultured with regular complete medium for two additional days in the 2.5 μg/μL puromycin. Lastly, the cells were washed and analyzed for protein expression with the Western blotting protein assay. 

### 4.7. Transient RNA Interference

Small interfering RNA (siRNA) duplexes targeting human Hes1 sequences and a scrambled siRNA were purchased from Sigma-Aldrich. Transfection of the siRNA duplexes was performed by TransIT-TKO Transfection Reagent (Takara, Kusatsu, Shiga, Japan) according to the manufacturer’s instructions.

### 4.8. Combination Studies

In the in vitro combination studies, cells were seeded in 96-well plates (2000 cells/well) and incubated at 37 °C for 24 h before addition. Then cells were treated with RO4929097, BKM120 and BEZ235. Cell viability was quantified with the CellTiter-Blue assay. Drug synergy was analyzed by calculating the combination index (CI) as a measure of the interaction between two drugs. The Combination Index (CI) was calculated according to the median-effect principle of the Chou and Talalay method, using the CalcuSyn software, version 2.1 (BioSoft, Great Shelford, Cambridge, UK). CI values are generated over a range of Fa levels, from 0.05–0.90 (5–90% growth inhibition). A CI of 1 indicates an additive effect between two agents, whereas a CI of <1 or >1 indicates synergism or antagonism, respectively [32].

### 4.9. Animal Studies

The mice were housed and cared for at the animal care facility of Toho University School of Medicine in accordance with the institution’s guidelines for the care and use of laboratory animals. The experimental protocol was approved by the Animal Research Committee, Toho University School of Medicine (ARC/TUSM-R16-14). We examined the antitumor efficacy of RO4929097 and BKM120 in intracranial xenografts, using GICs. Nude (nu/nu) 6–8 week-old mice (*n* = 24) were purchased from Charles River Co. (Japan). In this study, we used a guide-screw system to implant 5 × 10^5^ GICT25 cells in DMEM/F-12 serum-free media (5 mL), as described previously [6], and then randomly divided the mice into four groups of six mice each. Starting on day 4 after the tumor cells were implanted, mice were treated by oral gavage with 10 mg/kg RO4929097 in methyl cellulose alone, 20 mg/kg BKM120 in methyl cellulose alone, a combination of RO4929097 and BKM120 in methyl cellulose or methyl cellulose alone (control) once a week for a total of 5 weeks. Mice were monitored daily and euthanized when they became moribund. At necropsy, all organs were analyzed grossly and microscopically to assess toxicity.

### 4.10. Statistical Analysis

The data were analyzed with the Student unpaired *t*–test. The results are presented as the mean of at least three independent experiments. Differences were considered significant at a *p* value of <0.05, in all comparisons.

## 5. Conclusions

Our data show that PTEN is an important mediator of GSI-induced attenuation of cell growth and suggest the presence of a regulatory circuit linking NOTCH signaling with the PI3K/PTEN/AKT pathway. This finding may yield new therapeutic strategies and indicates that both pathways should be simultaneously inhibited in order to improve therapeutic efficacy in human GBMs. 

## Figures and Tables

**Table 1:** PTEN expression is required for the GSI response in cell growth inhibition. (**a**) PTEN knockdown decreased the response to GSI in PTEN–wild-type GIC. (**b**) PTEN expression increased the response to GSI in PTEN-mutant GIC.

IC_50_	GICT25	shPTEN
**(a)**		
DAPT (µM)	7.69	19.8
BMS-708163 (µM)	0.55	2.00
RO4929097 (µM)	0.96	4.67
**(b)**		
DAPT (µM)	13.50	5.78
BMS-708163 (µM)	1.82	0.84
RO4929097 (µM)	2.21	1.11

**Table 2:** Combination Index of GSI and PI3K inhibitor in GICs cells in vitro.

Reagents	CI Values at ED50
BKM120/GSI (2:1)	0.47
BEZ235/GSI (1:5)	0.58

## Supplementary Materials

The following are available online at http://www.mdpi.com/2072-6694/11/1/121/s1, Figure S1: γ-Secretase inhibitors. DAPT, BMS-708163 and RO4929097 were purchased from Selleck Chemicals.
