# Supplementary material for: The Oncogene Addiction Switch from NOTCH to PI3K Requires Simultaneous Targeting of NOTCH and PI3K Pathway Inhibition in Glioblastoma
Source: Cancers (Basel). 2019 Jan 20;11(1):121. doi: 10.3390/cancers11010121 (PMC6356490; doi:10.3390/cancers11010121)
Supplement: Supplementary file 1 [file cancers-11-00121-s001.pdf]

cancers

MDPI

Supplementary Material: The Oncogene Addiction Switch from NOTCH to PI3K Requires Simultaneous Targeting of NOTCH and PI3K Pathway Inhibition in Glioblastoma

Norihiko Saito, Nozomi Hirai, Kazuya Aoki, Ryo Suzuki, Satoshi Fujita, Haruo Nakayama, Morito Hayashi, Keisuke Ito, Takatoshi Sakurai and Satoshi Iwabuchi

DAPT

M.Wt: 432.46

Formula: $C_{23}H_{26}F_2N_2O_4$ C_{23}

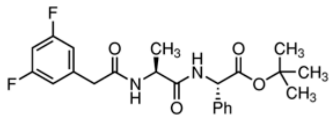

BMS-708163

M.Wt: 520.88

Formula: $C_{20}H_{17}ClF_4N_4O_4S$

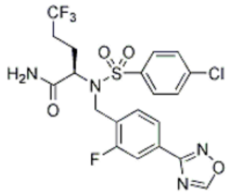

RO4929097

M.Wt: 469.4

Formula: $C_{22}H_{20}F_5N_3O_3$

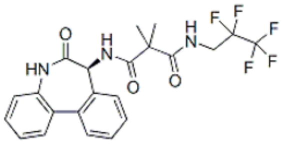

Figure S1. γ-Secretase inhibitors. DAPT, BMS-708163 and RO4929097 were purchased from Selleck Chemicals. For in vitro use, all inhibitors were dissolved in dimethyl sulfoxide (DMSO; Sigma-Aldrich) to a concentration of 10 mmol/L, stored at −20°C and further diluted to an appropriate final concentration in DMEM/F12 medium at the time of use. DMSO in the final solution did not exceed 0.1% (v/v).
